# Expression and Distribution Pattern of Aquaporin 4, 5 and 11 in Retinas of 15 Different Species

**DOI:** 10.3390/ijms17071145

**Published:** 2016-07-16

**Authors:** Barbara Amann, Kristina J. H. Kleinwort, Sieglinde Hirmer, Walter Sekundo, Elisabeth Kremmer, Stefanie M. Hauck, Cornelia A. Deeg

**Affiliations:** 1 Institute for Animal Physiology, Department of Veterinary Sciences, Ludwig-Maximilians-University, Veterinärstraße 13, D-80539 Munich, Germany; baerbl.amann@tiph.vetmed.uni-muenchen.de (B.A.); K.Kleinwort@tiph.vetmed.uni-muenchen.de (K.J.H.K.); Sieglinde.Hirmer@tiph.vetmed.uni-muenchen.de (S.H.); 2 Clinic for Ophthalmology, University Clinic Gießen und Marburg GmbH, Marburg, Baldingerstrasse, D-35033 Marburg, Germany; sekundo@med.uni-marburg.de; 3 Institute of Molecular Immunology, Helmholtz Zentrum München, German Research Center for Environmental Health (GmbH), Marchioninistraße 25, D-81377 München, Germany; krexit-hmgu@outlook.de; 4 Research Unit Protein Science, Helmholtz Zentrum München, German Research Center for Environmental Health GmbH, Heidemannstr. 1, D-80939 München, Germany; hauck@helmholtz-muenchen.de; 5 Experimental Ophthalmology, Philipps University of Marburg, Baldingerstrasse, D-35033 Marburg, Germany

**Keywords:** aquaporin 4, aquaporin 5, aquaporin 11, AQP, retina, Muller glia, water channels, glial fibrillary acidic protein, glutamine synthase, immunohistochemistry, tissue water flux, osmotic control

## Abstract

Aquaporins (AQPs) are small integral membrane proteins with 13 members in mammals and are essential for water transport across membranes. They are found in many different tissues and cells. Currently, there are conflicting results regarding retinal aquaporin expression and subcellular localization between genome and protein analyses and among various species. AQP4, 7, 9 and 11 were described in the retina of men; whereas AQP6, 8 and 10 were earlier identified in rat retinas and AQP4, 5 and 11 in horses. Since there is a lack of knowledge regarding AQP expression on protein level in retinas of different animal models, we decided to analyze retinal cellular expression of AQP4, 5 and 11 in situ with immunohistochemistry. AQP4 was detected in all 15 explored species, AQP5 and AQP11 in 14 out of 15. Interestingly, AQP4 was unambiguously expressed in Muller glial cells, whereas AQP5 was differentially allocated among the species analyzed. AQP11 expression was Muller glial cell-specific in 50% of the animals, whereas in the others, AQP11 was detected in ganglion cell layer and at photoreceptor outer segments. Our data indicate a disparity in aquaporin distribution in retinas of various animals, especially for AQP5 and 11.

## 1. Introduction

Aquaporins (AQPs) are integral membrane proteins forming transmembrane channels that are indispensable for water transport across the cell. They are found in many species and are ubiquitously expressed [1]. This protein family currently consists of 13 members and facilitates water transport across the plasma membranes of cells in response to osmotic stimuli [2]. The 13 aquaporins were all reported to be expressed in the eye [3,4]. The AQPs are grouped into three clusters: the classical aquaporins (0, 1, 2, 4, 5, 6, 8 and 9), the aquaglyceroporins (3, 7, 9 and 10) and the unorthodox aquaporins (11 and 12) [5]. All aquaporins allow water movement through facilitated diffusion under the control of local osmotic gradients [5]. Members of the aquaglyceroporin group are additionally permeable to glycerol and other small solutes [5]. The unorthodox aquaporins are less understood so far and differ at least in their structure and subcellular distribution pattern from the other groups [5].

Since the retina belongs to the central nervous system, damage of retinal cells results in irreversible damage to the retinal architecture and subsequent loss of vision. Therefore, cellular homeostasis and adequate regulation of substrate transport and cell volume is mandatory to ensure proper visual function. Changes in water channel expression can result in significant damage to the central nervous system (CNS). In cytotoxic brain edema, an osmotic gradient develops through hyponatremia and brain cells subsequently swell from water influx through vessels [6]. Intracellular volume increases, especially in astrocytes, where AQP4 is the dominant plasma cell membrane water channel [6]. Inhibition of AQP4 was beneficial in a mouse model of cytotoxic edema [6]. In contrast, in vasogenic brain edema that develops after damage to the blood-brain barrier—followed by interstitial water inflow into the brain through a hydrostatic gradient from blood to brain—AQP4 expression reduces brain edema [7]. There is ongoing research about potential therapeutic AQP4 inhibitors and activators, but to date there are no validated substances for therapy [2]. 

In the eye, similar differences of AQP4 function were shown. AQP4 plays an important role in retinal water homoeostasis [8]. Whereas AQP4 inhibition was neuroprotective in a retinal ischemia model [9], streptozotocin-induced diabetic retinopathy and light-induced retinal damage worsened without AQP4 expression [10,11]. Therefore, whether AQP4 is protective or destructive in certain conditions depends on the exact pathophysiology and on a potential different cellular expression pattern in physiology and disease. 

In a former study, we showed a difference in AQP4 protein levels as well as a different localization pattern in a spontaneous model of recurrent uveitis in horses [12]. In uveitic retinas, AQP4 increased and displaced from Muller glial cells to cell nuclei, where it was intensely expressed at the outer nuclear layer [12]. AQP5 was also expressed in physiological equine Muller glial cells, with strong enrichments in Muller cell secondary processes significantly decreased in uveitis [12]. This indicated significant changes in aquaporin function in this spontaneous inflammatory retinal disease, from our point of view. Interestingly, we recently identified AQP11 as the most prominently downregulated aquaporin in retinas in cases of uveitis, pointing to an interesting role of this unorthodox aquaporin in retinal function [13]. 

Aquaporin expression was shown at the transcriptome level for all aquaporins in the human eye, but there is still a lack of knowledge of protein expression level in different retinal cell types in many animal species and animal models. Therefore, we decided to analyze the distribution pattern of AQP4, 5 and 11 with immunohistochemistry. For the detection of AQP11 we used a novel monoclonal anti-AQP11 antibody, generated to bind a conserved linear epitope of mammalian aquaporin 11 [13].

## 2. Results

### 2.1. In Different Species, Aquaporin 4 (AQP4) is Predominantly Expressed at Retinal Muller Glial Cells

**Figure 1:**
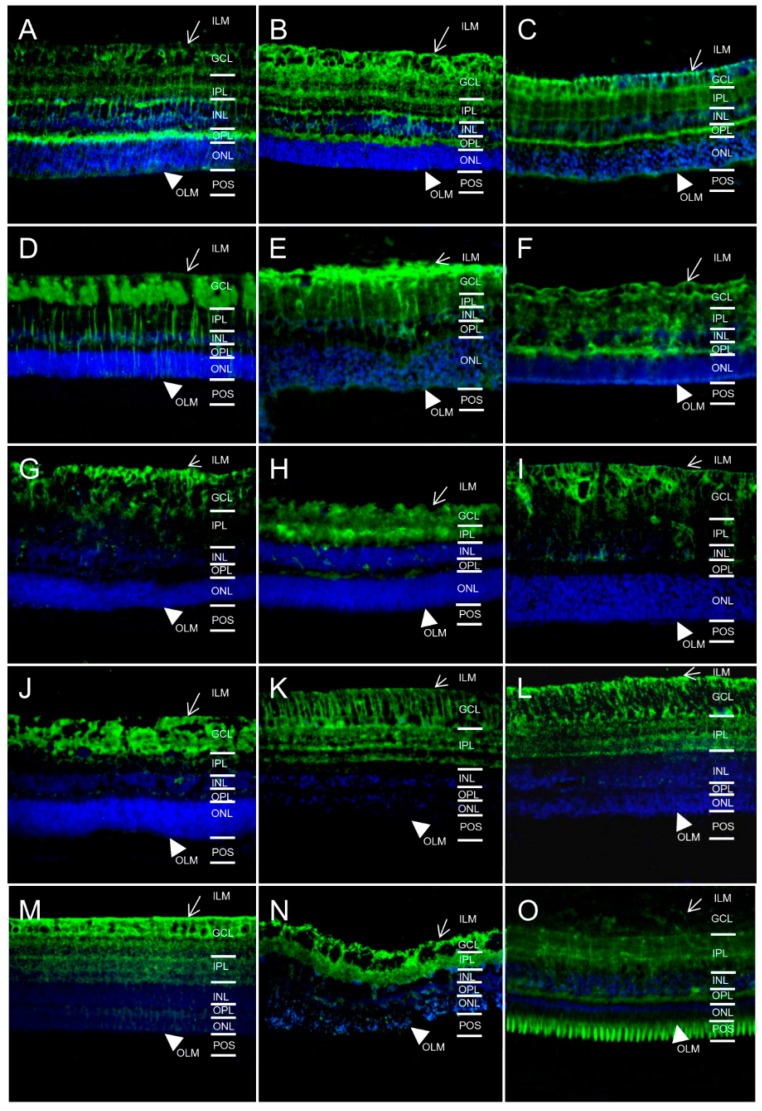
Predominant expression of aquaporin 4 (AQP4, green) at Muller glial cells in retinas of different species (magnification ×40). ILM, inner limiting membrane (arrow); GCL, ganglion cell layer; IPL, inner plexiform layer; INL, inner nuclear layer; OPL, outer plexiform layer; ONL, outer nuclear layer; OLM, outer limiting membrane (arrowhead); POS, photoreceptor outer segments. (**A**) mouse; (**B**) rat; (**C**) guinea pig; (**D**) horse; (**E**) cow; (**F**) sheep; (**G**) deer; (**H**) pig; (**I**) cat; (**J**) dog; (**K**) pigeon; (**L**) chicken; (**M**) pheasant; (**N**) sturgeon; (**O**) char. Cell nuclei: blue ((4′,6-diamidino-2-phenylindole) DAPI).

Since AQP4 is the main water channel protein in the retina of mice and men, we first analyzed AQP4 expression and cellular localization pattern in different species using immunohistochemistry. Interestingly, of 15 different species tested, all were positive for AQP4 expression in Muller glia (Figure 1, AQP4 green). AQP4 expression in Muller glial cells is polarized with the strongest distribution at Muller glial cell endfeet and inner limiting membrane (ILM), fading towards the outer limiting membrane. In some animals, additional expression at other sites was detectable, whereas in horses (Figure 1D), cows (Figure 1E), deer (Figure 1G), dog (Figure 1J), pigeon (Figure 1K), chicken (Figure 1L), pheasant (Figure 1M) and sturgeon (Figure 1N) the expression was limited to Muller glial cells. In mice (Figure 1A), rats (Figure 1B), guinea pigs (Figure 1C), sheep (Figure 1F), pigs (Figure 1H) and char (Figure 1O), AQP4 was additionally expressed at the outer plexiform layer. Further, a strong expression was detected at photoreceptor outer segments (POS) in char (Figure 1O).

### 2.2. AQP5 Expression Pattern Varies in Retinas of Different Species

**Figure 2:**
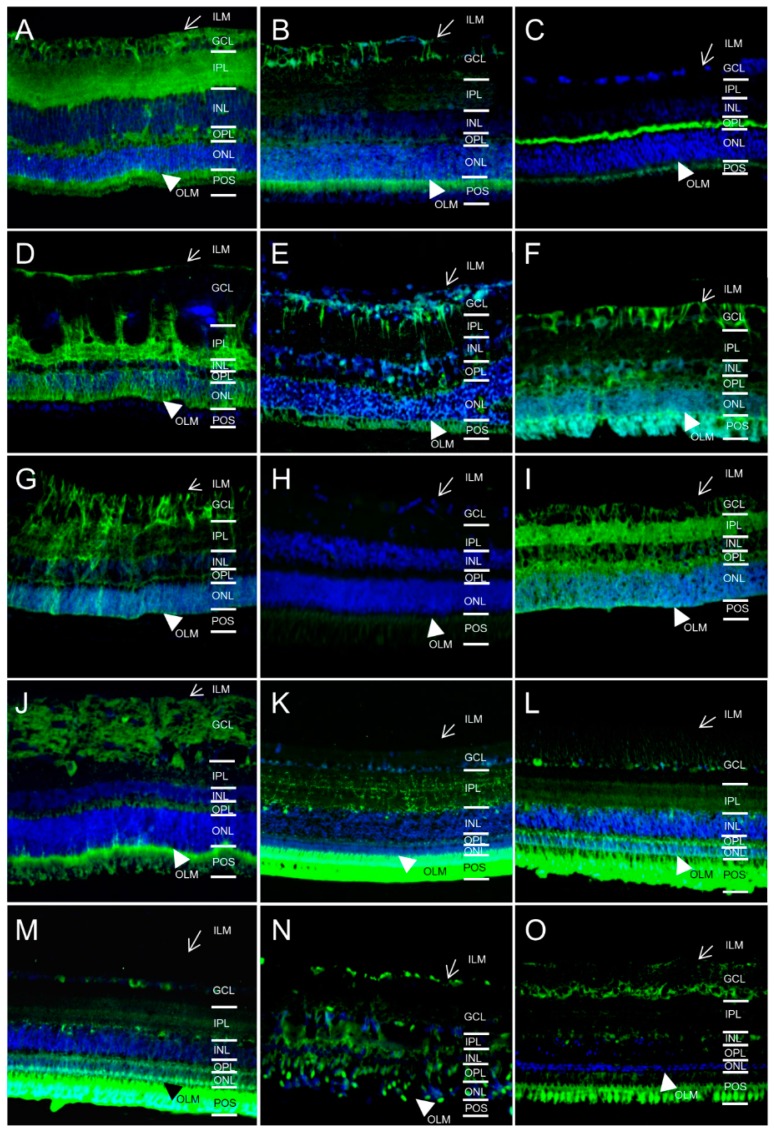
Diverse distribution of AQP5 (green) in retinas of different species (magnification ×40). ILM, inner limiting membrane (arrow); GCL, ganglion cell layer; IPL, inner plexiform layer; INL, inner nuclear layer; OPL, outer plexiform layer; ONL, outer nuclear layer; OLM, outer limiting membrane (arrowhead); POS, photoreceptor outer segments. (**A**) mouse; (**B**) rat; (**C**) guinea pig; (**D**) horse; (**E**) cow; (**F**) sheep; (**G**) deer; (**H**) pig; (**I**) cat; (**J**) dog; (**K**) pigeon; (**L**) chicken; (**M**) pheasant; (**N**) sturgeon; (**O**) char. Cell nuclei: blue (DAPI).

Additionally, we screened for AQP5 expression in respective species, because little is known about AQP5 expression in the retina so far. In former studies, we already detected and confirmed presence of AQP5 channels in horse retina [12,14]. In this study, we provide evidence of AQP5 in retinas of all species examined besides the pig (Figure 2). But in contrast to AQP4, the distribution pattern of AQP5 varied considerably among the animals. AQP5 was located at the outer limiting membrane of all AQP5 positive species. Further, in some animals AQP5 was expressed at retinal Muller glial cells like in mice (Figure 2A), rats (Figure 2B), horses (Figure 2D), cows (Figure 2E), sheep (Figure 2F), deer (Figure 2G), cat (Figure 2I) and dogs (Figure 2J). In the examined birds (Figure 2L,M), distinct expression was detectable in ganglion cell layer (GCL) and in inner and outer plexiform layer (IPL/OPL). Further, AQP5 was highly positive at photoreceptor outer segments (POS) of these birds (Figure 2L,M) and also in mice (Figure 2A), rats (Figure 2B), cows (Figure 2E), sheep (Figure 2F), dog (Figure 2J) and char (Figure 2O). In mice (Figure 2A), horses (Figure 2D) and cats (Figure 2I), AQP5 was additionally found in the entire inner plexiform layer (IPL). Therefore, AQP5 distribution varied considerably among retinas of these diverse creatures, indicating a different function in these animals and animal groups (like birds).

### 2.3. AQP11 Is Primarily Located at Retinal Muller Glial (RMG) Cells and Photoreceptor Outer Segments in Most Retinas Examined

**Figure 3:**
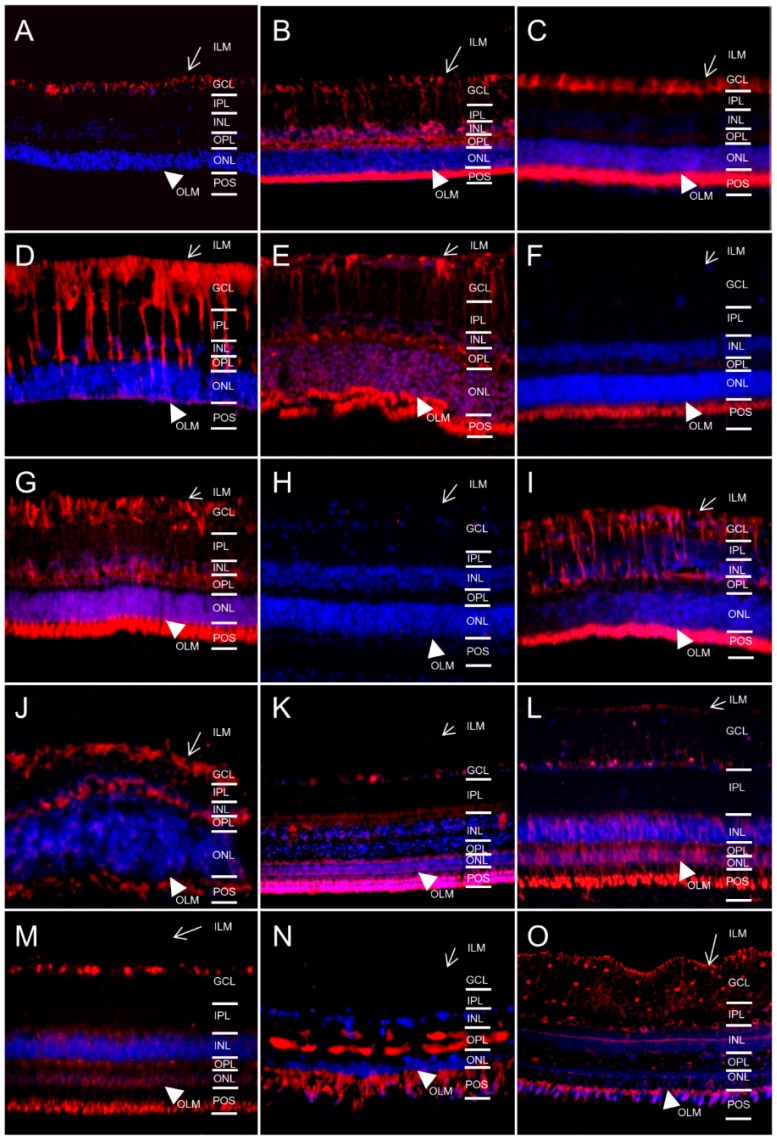
Expression patterns of AQP11 (red) in retinas of different species (magnification ×40). ILM, inner limiting membrane (arrow); GCL, ganglion cell layer; IPL, inner plexiform layer; INL, inner nuclear layer; OPL, outer plexiform layer; ONL, outer nuclear layer; OLM, outer limiting membrane (arrowhead); POS, photoreceptor outer segments. (**A**) mouse; (**B**) rat; (**C**) guinea pig; (**D**) horse; (**E**) cow; (**F**) sheep; (**G**) deer; (**H**) pig; (**I**) cat; (**J**) dog; (**K**) pigeon; (**L**) chicken; (**M**) pheasant; (**N**) sturgeon; (**O**) char. Cell nuclei: blue (DAPI).

Whereas AQP4 and 5 are assigned to the classical aquaporins, which primarily transport water [14], AQP11 belongs to an only distantly related aquaporin family [14]. We confirmed our earlier findings [13] of Muller glia-specific expression of AQP11 in mice (Figure 3A) and horses (Figure 3D). Interestingly, of 15 species tested, 8 were also positive for AQP11 expression (Figure 3, red) in Muller glia (mice (Figure 3A), rats (Figure 3B), guinea pigs (Figure 3C), horses (Figure 3D), cows (Figure 3E), deer (Figure 3G), cats (Figure 3I) and dogs (Figure 3J)).

**Figure 4:**
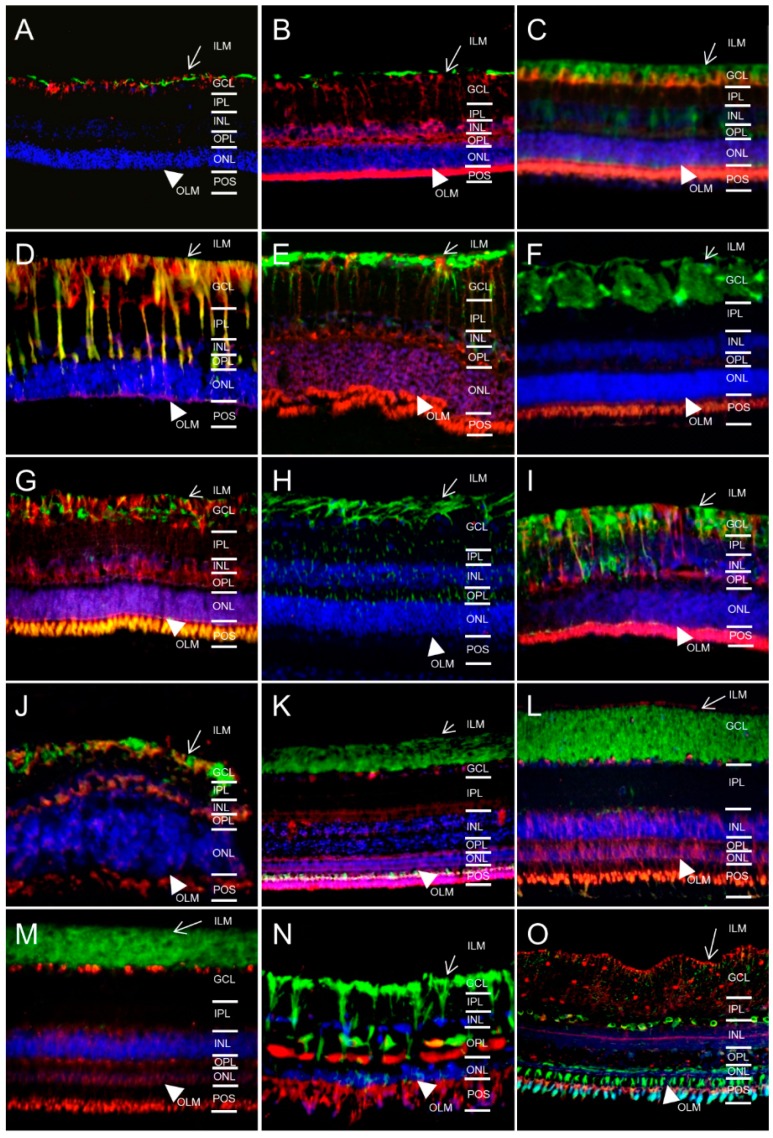
Overlay (yellow) of AQP11 (red) with Muller glial cell marker glial fibrillary acidic protein (GFAP, green; magnification ×40). ILM, inner limiting membrane (arrow); GCL, ganglion cell layer; IPL, inner plexiform layer; INL, inner nuclear layer; OPL, outer plexiform layer; ONL, outer nuclear layer; OLM, outer limiting membrane (arrowhead); POS, photoreceptor outer segments. (**A**) mouse; (**B**) rat; (**C**) guinea pig; (**D**) horse; (**E**) cow; (**F**) sheep; (**G**) deer; (**H**) pig; (**I**) cat; (**J**) dog; (**K**) pigeon; (**L**) chicken; (**M**) pheasant; (**N**) sturgeon; (**O**) char. Cell nuclei: blue (DAPI).

There was a slight difference in the Muller glia cell expression (Figure 4, overlays with astrocyte and retinal Muller glial cell marker glial fibrillary acidic protein (GFAP)) patterns between a prominent expression expanding along Muller cell bodies until the outer limiting membrane as seen in horses (Figure 4D and [13]) and an expression mainly limited to Muller glial endfeet at the inner limiting membrane as seen in mice (Figure 4A). AQP11 positive retinas of other animals showed a completely different pattern with labeling of photoreceptor outer segments only in sheep (Figure 4F) or multiple expression sites like ganglion cells (GCL), outer limiting membrane (OLM) and photoreceptor outer segments (POS) as in pigeons (Figure 4K), chickens (Figure 4L), pheasant (Figure 4M), char (Figure 4O). In sturgeon, besides POS, a part of the outer plexiform layer (OPL) was AQP11 positive (Figure 4N).

**Figure 5:**
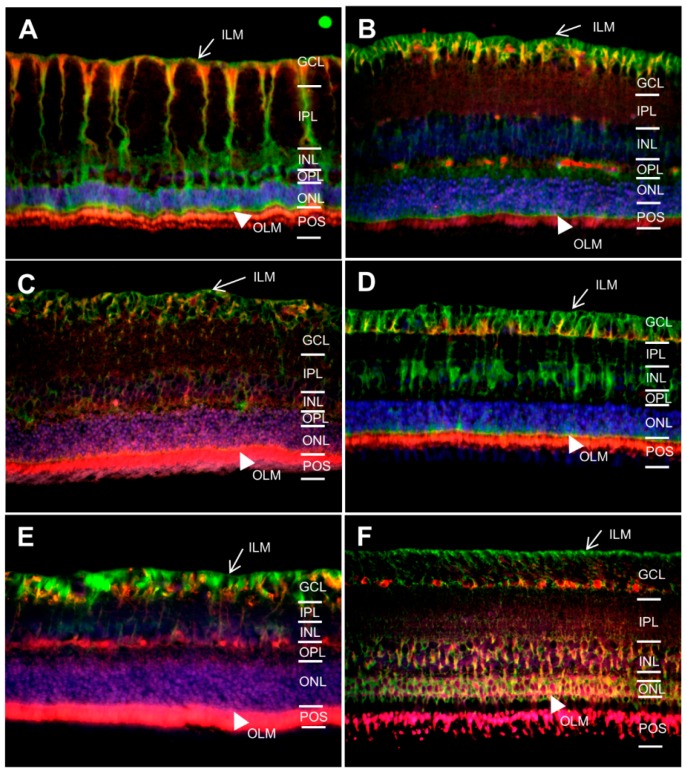
Overlay (yellow) of AQP11 (red) with Muller glial marker glutamine synthase (GS, green; magnification ×40). ILM, inner limiting membrane (arrow); GCL, ganglion cell layer; IPL, inner plexiform layer; INL, inner nuclear layer; OPL, outer plexiform layer; ONL, outer nuclear layer; OLM, outer limiting membrane (arrowhead); POS, photoreceptor outer segments. (**A**) horse; (**B**) mouse; (**C**) rat; (**D**) guinea pig; (**E**) cat; (**F**) chicken. Cell nuclei: blue (DAPI).

For species with AQP11 expression at RMG cells, we used an additional Muller glial marker (glutamine synthase, GS green) to confirm respective AQP11 localization (Figure 5).

Specificity of primary antibody bindings was controlled with respective isotype controls (representative stainings in supplemental Figure S1, mouse isotype control green).

## 3. Discussion

Water transport is crucial in the eye, especially since it is a widely avascular tissue that depends on transport of nutrients, fluids and ions through diffusion over membranes [4,8]. Aquaporins are specialized channels that most rapidly transport water and other small solutes in many tissues. The 13 currently-known members of the aquaporin family detected in mammals are differentially distributed in many tissues and additionally vary in their subcellular localization [1,2]. In a complex organ like the eye, dissimilar aquaporins are expressed by cells of different tissues [5]. For example, aquaporin 0 (formerly named MIP) is very important for the transparency of the lens and ensures water removal from the lens fibers, that have a high protein and low water content [4,15]. In the retina, aquaporin 4 was described as predominant water channel in man [16] and mice [4] and is expressed in Muller glial cells [16]. Muller cells are important modulators of neuronal activity through modifications of the concentrations of ions, neurotransmitters and other neuroactive substances within the extracellular space between the inner and the outer limiting membrane [16]. For extracellular homeostasis, Muller cells buffer extracellular potassium (K^+^) via inward rectifying K^+^ channels (Kir channels) [16]. There is an ongoing discussion of whether the involvement of both AQP4 and Kir channels in regulating the extracellular environment in the brain and retina are functionally dependent [16,17,18]. Nevertheless, AQP4 maintains the integrity of the blood-brain barrier and plays an important role in maintaining the homeostasis of water and ions in the CNS [19]. But AQP4 can contribute to the formation [20] as well as to the resolution [7] of brain edema. 

In the retina, there were also substantial functional differences noted, because AQP4 can be neuroprotective or neurodestructive, dependent on the type of injury and experimental model used. Inhibition of AQP4 was neuroprotective in retinal ischemia [9], but it significantly exacerbated diabetic retinopathy [10] and light-induced retinal damage [11]. In our natural model of neuroinflammation in the retina, spontaneous equine recurrent uveitis (ERU), we detected an increase of the total protein amount of AQP4 [12]. But interestingly, AQP4 expression changed its localization in ERU from Muller cells to a strong circular expression in the outer nuclear layer and rarely in the inner nuclear layer, whereas its expression in Muller cell trunks almost disappeared [12]. The question is how gliotic Muller cells transport water without AQP4. Earlier, we analyzed retinal membrane proteins of healthy horses and ERU cases with differential proteome analyses [21]. In this study, we identified protein expression of AQP4, 5 and 11 in retina of healthy horses [21]. Additionally, we performed experiments to clarify the cellular expression pattern of AQP4 and 5 in horse retinas and identified expression of AQP4 at physiological levels in Muller cells [12]. AQP5 was detected with immunohistochemistry at a considerable level throughout the retina of horses with enrichments in the IPL and the outer nuclear layer [12] and clearly co-localized with Muller cell marker glutamine synthase [12].

This finding was in contrast with published RT-PCR data analyzing rat ocular tissues, assigning AQP5 to cornea, lens, iris and ciliary body, but did not detect AQP5 in the retina [22]. It was earlier noted that gene expression data can markedly differ from actual protein expression sites and this was also shown for aquaporins [23]. In the study conducted here, we could again confirm expression of AQP4 and 5 in equine Muller cells. Whereas AQP4 was detected in all retinas examined with a clear predominant retinal Muller glial cell expression, this was completely different for AQP5. AQP5 (as well as 11) could not be detected in pig retinas (Figure 2, Figure 3 and Figure 4H, respectively) and was found to be differentially expressed in the AQP5 positive retinas of the 14 other species analyzed. Whereas AQP5 localization was similar at the outer limiting membrane of all AQP5 positive retinas of the different species examined, the further expression patterns showed a great variety of AQP5 distribution in retina (GCL, IPL, OPL, POS). This probably indicates a considerate functional difference of AQP5 in these retinas. Further studies are needed to clarify the respective roles of AQP5 channels in the retina, especially since AQP5 was reported in corneal epithelium, lacrimal gland and retinal pigment epithelium in human and rat eyes before, but not in the retina [8]. Our data in the rat retina are controversial to this, but exact AQP expression is controversial for many tissues, e.g., in salivary glands [24]. This is partly the result of insufficient analyses tools for protein expression, e.g., a lack of good antibodies [25,26] and additionally, a marked difference of cellular localization between mRNA data and immunocytochemical analyses was described for aquaporins [23]. Therefore, analysis of aquaporin expression should be done on protein level to get more secure results for cellular localization. We used a cross-reactive AQP5 antibody generated to a 17-amino acid sequence in the cytoplasmic region of rat AQP5 that is commercially available and suited for immunohistochemistry. Since we used three different primary rabbit antibodies to AQP4, 5 and GFAP with the same secondary antibody and obtained very different results for each protein per species, we rule out unspecific binding of the antibodies. Additionally, we conducted isotype control stainings and examined all sections for autofluorescence that is often found in POS of retina sections (supplemental Figure S1). For the detection of aquaporin 11 in retinal sections, we recently created a cross-reactive validated rat monoclonal antibody to AQP11 [13].

With this antibody, we confirmed strong expression of AQP11 in equine retina [21] at Muller cell membranes [13]. Interestingly, this is in contrast to multiple other organs, where the superaquaporin AQP11 was allocated intracellularly and co-localized with rough endoplasmatic reticulum [25,27]. But AQP11 expression was also shown to be located at endfeet of Muller cells in the inner limiting membrane of human retina [27], confirming various expression of AQP11 in different cell types and organs. In this study, we could—for the first time—analyze AQP11 expression on protein level in retinas in a wide variety of species. Interestingly, in all retinas besides the pig, AQP11 was detectable. Thereby, two major patterns of AQP11 expression were evident, a Muller glial cell specific one in 50% of the analyzed species and a second expression pattern, that was predominantly found in birds and fish. In these animals, AQP11 was distributed in the ganglion cell layer and in the photoreceptor outer segments. The difference in AQP distribution indicates functional differences of respective aquaporins in different animal species and also dissimilarities in water and solute transport in different retinas. This knowledge is important, because large animal models gain more significance in ophthalmology research [28]. To choose the right animal models for respective research, profound insight about anatomical and physiological differences and conformities are needed.

To gain such knowledge, we used proteome analyses to identify water channel expression in different animal models, to clarify the function of water channels in physiology and different pathophysiological conditions, especially in neuroinflammation and edema formation. Our proteome analyses of healthy and ERU retinas did not indicate further AQPs in healthy horse retina or ERU condition besides AQP4, 5 and 11 [21] and recent data on isolated mouse Muller cells [29] and unpublished observations support our assumption that these are the dominant aquaporins expressed in the retina. Therefore, in this study, we only analyzed protein expression of these aquaporins in retinas of different species in situ. But further studies will probably clarify the existence and potential role of other aquaporins in the retina. Additionally, we cannot entirely rule out other pathways/channels capable of water transport in retinal pathophysiology, especially when aquaporins disappear from the retina or are dislocated during retinal injury. 

## 4. Materials and Methods

### 4.1. Animal Eye Specimen

For this study, eyes of 15 different animal species without known history or clinical evidence of eye diseases were used. No experimental animals were used in this study. All animals died from causes unrelated to this study. Eyes were sampled from euthanized patients of veterinary faculty, at local slaughterhouses or were huntable deer and sampled under game law (by author Barbara Amann, hunter) [30]. Research was approved by local government, collection and use of eyes from slaughtered animals that were killed due to a research-unrelated cause was approved for purposes of scientific research by the appropriate board of the veterinary inspection office Munich, Munich, Germany (Permit number: 8.175.10024.1319.3).

### 4.2. Preparation for Immunohistochemistry

Posterior eyecups were immediately immersion-fixed with Bouin’s solution (Sigma Aldrich, Taufkirchen, Germany). Fixation was followed by dehydration in ascending alcohol series [31]. After dehydration, the sections were embedded in paraffin (Microm, Walldorf, Germany). Tissue sections were cut at 8 µm and mounted on coated slides (Super Frost Plus, Medite, Burgdorf, Germany), deparaffinized and rehydrated.

### 4.3. Immunohistochemical Detection of Aquaporins

Tissue sections were deparaffinized with xylol. Then, sections were rehydrated in descending alcohol series. Heat antigen retrieval was performed in citrate buffer pH 6.0 followed by EDTA buffer pH 8.0 (0.1 M EDTA—Dinatriumsalz—Dihydrat, 0.1 M NaOH) at 99 °C for 15 min each. To prevent unspecific antibody binding, sections were blocked with 1% bovine serum albumin in Tris buffered saline-tween (TBS-T; 150 mM NaCl, 10 mM Tris, pH 7.2, 0.1% Tween 20) containing 5% normal goat serum for 40 min at room temperature. Then, sections were incubated with primary antibodies to AQP4, 5, 11 or glial fibrillary acidic protein (GFAP). The following antibodies were used: rabbit anti-AQP4 antibody (Alomone, Duisburg, Germany; dilution 1:200), rabbit anti-AQP5 antibody (Calbiochem, Darmstadt, Germany; dilution 1:500) and a novel mouse IgG1 monoclonal antibody clone 8H9 to AQP11, generated towards a linear 12mer epitope of horse AQP11 in a widely conserved sequence [13]. Respective novel anti-AQP11 antibody was shown to cross-react with mouse and rat AQP11 with pre-incubation with immunization peptide specific for AQP11 and an irrelevant peptide. Pre-incubation with AQP11 omitted primary antibody binding [13]. For detection of Muller glial cells and astrocytes, we took rabbitanti-GFAP (Dako-Cytomation, Hamburg, Germany; dilution 1:1000) and mouse anti-glutamine synthase (BD Biosciences, Heidelberg, Germany; dilution 1:1500). As negative controls, control slides were stained with isotype controls to all primary antibodies used in this study. For the evaluation of autofluorescence present in respective sections, isotype control to anti-mouse IgG Alexa Fluor 488 is exemplarily shown in supplemental Figure S1, since autofluorescence is especially present in green in retinas.

Primary antibodies were incubated at 4 °C overnight, followed by a washing step with TBS-T. Then, sections were incubated with respective secondary antibodies. We used anti-mouse IgG Alexa Fluor 568, anti-mouse IgG Alexa Fluor 488 or anti-rabbit IgG Alexa Fluor 488 (both Invitrogen, Karlsruhe, Germany; dilution: 1:500) respectively for 30 min at room temperature. Afterwards, cell nuclei were counter-stained with 4′,6 diamidino-2-phenylindole (DAPI; Invitrogen; dilution: 1:1000). Finally, sections were mounted with glass cover slips using Dako fluorescence mounting medium (Dako-Cytomation). Fluorescence stainings were recorded with Axio Imager M1 (Zeiss, Göttingen, Germany) and visualized with the Axio Vision 4.8 software (Zeiss).

## 5. Conclusions

Aquaporins are crucial for water transport in the retina, a tissue that is widely avascular and dependent on water and particle transport through cell membranes. The prerequisite of studying their function is the detection of aquaporin expression on protein level in situ. We analyzed expression patterns of AQP4, 5 and 11, the major retinal aquaporins according to our proteome analyses in various animal species. AQP4 was detected in all 15 species and is predominantly expressed in retinal Muller glial cells. AQP5 and AQP11 were detected in all retinas examined beside the pig. AQP5 showed the most varied expression patterns among different species and was found in ganglion cell, inner/outer plexiform layers and photoreceptor outer segments in very unique distribution patterns. Additionally, we confirmed the presence of AQP11 in many retinas with two different expressions. Either AQP11 was predominantly expressed at retinal Muller glial cells (8 from 15 species) or it was widely expressed at many different retinal localizations without Muller glial cells. Our data confirm AQP4, 5 and 11 expression in retinas and point to functional differences of aquaporins in different cells and tissues through their modified localizations.

## Supplementary Materials

Supplementary materials can be found at http://www.mdpi.com/1422-0067/17/7/1145/s1.

## Abbreviations

AQP	Aquaporin
CNS	Central nervous system
DAPI	4′,6 Diamidino-2-phenylindole
GFAP	Glial fibrillary acidic protein
IgG	Immunoglobulin G
MIP	Major intrinsic protein
