# Supplementary material for: Expression and Distribution Pattern of Aquaporin 4, 5 and 11 in Retinas of 15 Different Species
Source: Int J Mol Sci. 2016 Jul 16;17(7):1145. doi: 10.3390/ijms17071145 (PMC4964518; doi:10.3390/ijms17071145)
Supplement: Supplementary file 1 [file ijms-17-01145-s001.pdf]

# Supplementary Materials: Expression and Distribution Pattern of Aquaporin 4, 5 and 11 in Retinas of 15 Different Species

**Barbara Amann, Kristina J. H. Kleinwort, Sieglinde Hirmer, Walter Sekundo, Elisabeth Kremmer, Stefanie M. Hauck and Cornelia A. Deeg**

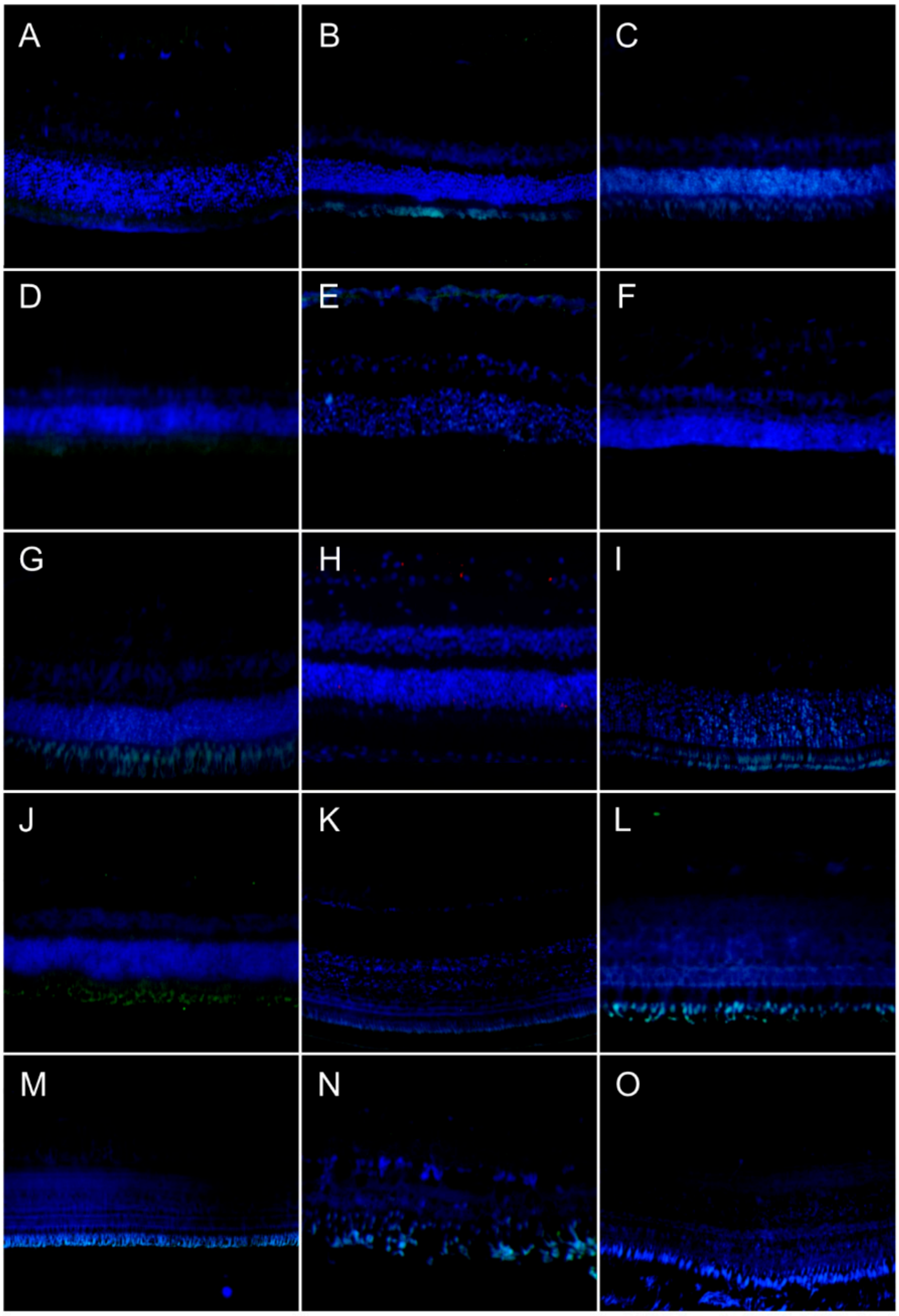


**Figure S1.** Mouse isotype control (green) stainings and autofluorescence in examined species (magnification ×40). (**A**) mouse; (**B**) rat; (**C**) guinea pig; (**D**) horse; (**E**) cow; (**F**) sheep; (**G**) deer; (**H**) pig; (**I**) cat; (**J**) dog; (**K**) pigeon; (**L**) chicken; (**M**) pheasant; (**N**) sturgeon; (**O**) char. Cell nuclei: blue ((4′,6-diamidino-2-phenylindole) DAP I) (DAPI).
